# A scientometric analysis of research trends, visualization, and emerging patterns in canine olfactory detection for cancer

**DOI:** 10.14202/vetworld.2024.1430-1434

**Published:** 2024-07-06

**Authors:** Fran Espinoza-Carhuancho, Franco Mauricio, Cesar Mauricio-Vilchez, Diego Galarza-Valencia, Julia Medina, Josmel Pacheco-Mendoza, Frank Mayta-Tovalino

**Affiliations:** 1 Bibliometrics, Evidence Evaluation and Systematic Review Group, Human Medicine Career, Universidad Cientifica del Sur, Lima, Peru; 2 Research, Innovation and Entrepreneurship Unit, Universidad Nacional Federico Villarreal, Lima, Peru; 3 Department of Academic, Faculty of Medical Technology, Universidad Nacional Federico Villarreal, Lima, Peru; 4 Vicerrectorado de Investigación, Universidad San Ignacio de Loyola, Lima, Peru

**Keywords:** canine olfactory, oncology, scientometric

## Abstract

**Background and Aim:**

Dogs can detect specific cancer odors with their exceptional sense of smell. This study aimed to conduct a scientometric analysis of canine olfactory detection in oncology, identifying trends, visualizations, and patterns.

**Materials and Methods:**

A retrospective observational study was conducted using a quantitative-scientometric approach. Scopus was comprehensively searched using terms related to canine olfactory detection in oncology. Documents indexed in Scival software (Elsevier) and published between 2013 and 2022 were included.

**Results:**

Claire M. Guest, Rob Harris, and Giuseppe Lippi authored significant academic work. Journals such as Journal of Breath Research and PLoS One rank highly in publications and citations due to significant citation ratios, according to CiteScore’s quartile-based impact analysis. According to Lotka’s and Bradford’s laws, a small group of authors and the Journal of Breath Research, respectively, dominate production in their fields.

**Conclusion:**

This analysis forms a solid base for future research on canine olfactory detection in oncology. The collaborative essence of this multidisciplinary field is emphasized by the key contributors and identified patterns, with journals in the Q1 and Q2 quartiles of CiteScore holding significant importance.

## Introduction

Efforts to find new, reliable, specific, and sensitive cancer detection methods are underway [1] to target early disease stages. Although over two long decades have passed since the debut of prostate-specific antigen in medicine, current diagnosis and detection techniques for prostate cancer remain suboptimal, relying predominantly on qualitative tests such as quantification and digital rectal examination [2]. Therefore, significant efforts are being made to find new cancer detection procedures, which should be theoretically reliable, specific, and sensitive enough to detect the early disease stages [1]. A dog with specialized odor detection training can diagnose histologically confirmed cancer with approximately 93.5% sensitivity and 91.6% specificity [3].

Tests for colon and breast cancer that detect diseases early are successful in identifying premalignant conditions [4, 5]. Revolutionary biomarkers generated by cancerous and healthy cells could significantly enhance the precision of established early detection tests, even permitting early detection in cases where it is presently unattainable [6, 7]. The concept of employing a dog’s olfactory abilities for cancer detection was suggested by Williams and Pembroke [7]. A patient consulted a doctor due to her dog’s unusual focus on a mole on her skin. A malignant melanoma was discovered as a pathological finding post-mole removal [8]. Numerous studies acknowledge dogs’ superior sense of smell compared to current artificial sensors. Dogs are globally employed for detecting chemicals in civilian, military, and medical contexts [9]. Although they are essential biological sensors responsible for protecting life and property as olfactory organs, the boundaries of their olfactory sensitivity remain unexplored by research. The olfactory detection threshold refers to the lowest concentration of an odor that an individual can identify and distinguish from an odorless sample [10]. This threshold can be further defined based on a performance criterion related to a detection task, such as the percentage of correct responses or true positives [11].

The research into a dog’s remarkable sense of smell has been relatively limited. The unique ability of canines to identify scents linked to cancer is transforming the field of oncology, opening new avenues for the diagnosis and treatment of this terrible disease [1]. This study aimed to conduct a scientometric analysis of canine olfactory detection in oncology, identifying trends, visualizations, and patterns.

## Materials and Methods

### Ethical approval

Because this study was based on open access data, it does not require permission or authorization from any ethics committee.

### Study period and location

The study was conducted in February 2024 at the Universidad San Ignacio de Loyola, Lima-Peru.

### Design and search strategy

A retrospective observational study with a quantitative-scientometric approach was conducted. An exhaustive search in Scopus was conducted using terms associated with canine olfactory detection in oncology. The following search formula was used: TITLE-ABS-KEY (“Dogs’ olfactory” OR “Olfactory detection” OR “dogs sniffing” OR “Canine olfaction” OR “Scent detection” OR “Sniffer dogs” OR “Detection dogs” OR “Scent hounds”) AND (“cancer” OR “Tumor” OR “Carcinoma” OR “Neoplasm” OR “Malignancy” OR “Growth” OR “Lesion” OR “Tumor” OR “Carcinogenesis” OR “Metastasis” OR “Oncology” OR “Neoplasms” OR “Carcinomas” OR “Neoplastic Processes” OR “Cancerous growth” OR “Malignant tumor” OR “Benign tumor” OR “Metastatic cancer”).

### Data selection and extraction

The documents indexed in Scival software (Elsevier) between 2013 and 2022 were included in the study. Prioritization was given to primary research papers, reviews, notes, letters, book chapters, short surveys, and editorials. One hundred and twenty-nine articles were identified in the search conducted on February 27, 2024, among which were 16 reviews, eight notes, six letters, four conference papers, three book chapters, and two short surveys. An editorial was also examined on the topic hand of the interest.

### Scientometric analysis

The data encompassed citation details, author collaborations, trends over time, and journal impact. Through scientific production analysis using scientometric techniques, the evolution over time, popular collaborations among researchers, and impact of journals in the field of canine olfactory detection in oncology were assessed. The results will be illustrated graphically for easier understanding and to bring out hidden trends.

## Results

Several authors were recognized for their significant scholarly contributions and relevance. Claire M. serves as an example. With a total of six scholarly papers, the latest published in 2021, a citation index of 116, and an average of 19.3 citations per publication, Guest led the list, while Rob Harris followed closely with five publications through 2021, each boasting a citation index of 20.2. Despite having published just three papers, Giuseppe Lippi’s h-index stood impressively high at 99 (Table-1).

**Table-1:** Scholarly output.

Name	Scholarly output	Most recent publication	Citations	Citations per publication	Field-weighted citation impact	h-index
Guest, Claire M.	6	2021	116	19.3	1.95	15
Harris, Rob	5	2021	101	20.2	2.04	8
Hackner, Klaus	4	2020	63	15.8	1	8
Pleil, Joachim D.	4	2020	131	32.8	1.11	38
Otto, Cynthia M.	4	2022	34	8.5	1.29	31
Rudnicka, Joanna	3	2015	168	56	1.7	12
Bomers, Marije Kristianne	3	2015	105	35	1.51	23
Walczak, Marta	3	2015	168	56	1.7	12
Essler, Jennifer L.	3	2021	32	10.7	1.61	11
Lippi, Giuseppe	3	2021	21	7	0.58	99

Several prominent journals rose to the top in terms of publications and citations. The Journal of Breath Research published 12 articles, received 298 citations, and achieved a citation-to-publication ratio of 24.8. PLoS One had six publications with a total of 250 citations, resulting in a high citation index per publication of 41.7 and a source-normalized impact per paper (SNIP) of 1253. Scientific reports achieved a high SNIP score of 1.312 despite fewer citations. With a citation index per publication of eight and a SCImago journal rank of 1609, lung cancer, which has fewer publications, exhibited a remarkable impact in lung cancer research (Table-2).

**Table-2:** Top 10 scientific journals.

Scopus source	Publications	Citations	Citations per publication	Source-normalized impact per paper	CiteScore 2022	SCImago journal rank
Journal of Breath Research	12	298	24.8	0.96	7.4	0.81
PLoS One	6	250	41.7	1.253	6	0.885
Scientific Reports	6	51	8.5	1.312	7.5	0.973
Frontiers in Veterinary Science	6	125	20.8	1.11	3.8	0.737
Journal of Veterinary Behavior: Clinical Applications and Research	5	127	25.4	0.875	4.2	0.57
Clinical Chemistry and Laboratory Medicine	3	27	9	1.461	10.4	1.266
Medical Hypotheses	2	32	16	0.749	7.4	0.619
BMC Cancer	2	48	24	1.166	6.8	1.137
Ecosphere	2	11	5.5	0.986	5	1.067
Lung Cancer	1	8	8	1.346	10.3	1.609

**Figure-1:**
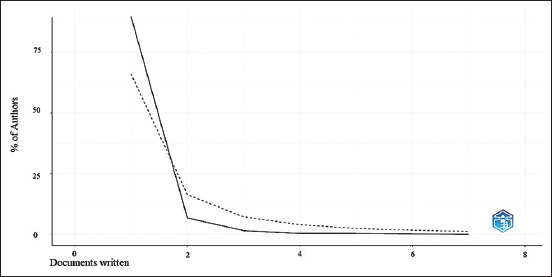
Author productivity by Lotka’s law.

To analyze papers written based on Lotka’s law, most authors have contributed a single paper (782 authors, representing 89.8% of the total); suggesting that some authors will contribute to most papers in a given field. In contrast, only 60 authors (6.9%) have contributed two papers, and 15 authors (1.7%) have contributed three papers. This distribution reflects a strong concentration of production in the hands of a select group of authors, whereas most authors make a limited contribution (Figure-1).

**Figure-2:**
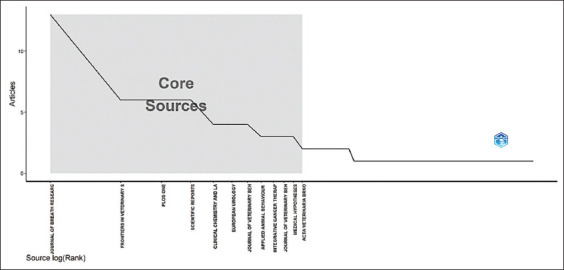
Core sources.

Bradford’s law revealed that the Journal of Breath Research topped the list in Zone 1, with 13 publications, cementing itself as the most productive journal in the dataset. This is followed by four other journals: Frontiers in Veterinary Science, PLoS One, Scientific Reports, and Clinical Chemistry and Laboratory Medicine, also in Zone 1, each with a significant number of publications. As we move toward Zone 2, a greater variety of journals is observed, each with a smaller number of publications than those in Zone 1. Despite the diverse journals in Zone 2, their contributions are less significant in the frequency of publications (Figure-2).

**Figure-3:**
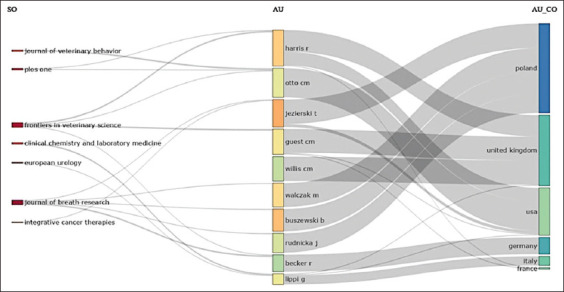
Three-field plot.

When analyzing the interactions between the three fields, Poland, the UK, and the US mainly led the scientific production on this topic. These countries were represented by authors Jezierski T, Walczak M, and Buszewski B, who published in journals such as Frontiers in Veterinary Science and Journal of Breath Research (Figure-3).

**Figure-4:**
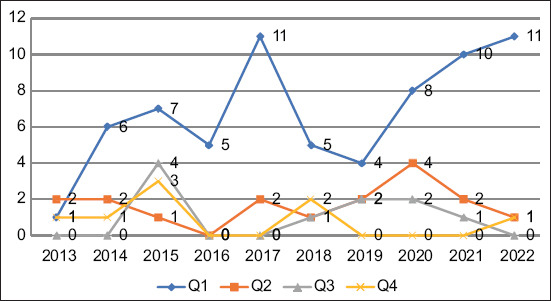
Publications by the CiteScore quartile.

The impact of the CiteScore quartile revealed distinctive patterns in the contributions of journals over the years. In the Q1 quartile, a significant contribution stood out with 68 publications over the years 2013–2022. This trend indicates that journals in this quartile have maintained a constant impact and have led in the CiteScore. Conversely, the Q2 quartile also presented a notable contribution, with 17 publications in the same period. Although this figure is lower than that in the Q1 quartile, it demonstrates a significant presence of journals that have maintained solid performance in this range. These results underline the importance of journals in the Q1 and Q2 quartiles in CiteScore, highlighting their predominant influence and contribution to the field analyzed (Figure-4).

## Discussion

According to studies [12, 13], dogs can distinguish breast cancer patient sweat samples from those of the control group. The dog showed 84% sensitivity and 81% specificity. In the double-blind tests, the specificity was recorded at 81%. The study confirms dogs’ accuracy in detecting breast cancer in seemingly healthy people. The results of this study align with those of earlier research [12, 13]. At early stages of cancer, dogs can distinguish cancerous from noncancerous tissue samples with exceptional precision. This ability has sparked great interest in the development of odor-detection devices that can replicate canine olfactory sensitivity [14].

In a study by Thuleau *et al*. [15] canines were trained to distinguish patients with breast cancer from those without the disease through their sense of smell using skin secretion samples. About 90.3% of the results were detected. Training dogs to detect cancer is a painstaking process involving the association with a specific odor with a reward, such as food or play [16]. By being repeatedly trained and rewarded, dogs can detect the distinct scent of cancer. Numerous medical centers globally have specialized training programs and canine cancer detection teams, thanks to this methodology. This discrimination ability could be invaluable for personalized medicine, where an accurate and rapid diagnosis is essential to determine the most appropriate treatment [17].

Like other diseases, cancer can alter body odors, according to Gouzerh *et al*. [18]. In the past several decades, scientists have intensely investigated the role of volatile organic compounds (VOCs) as cancer indicators. Two hundred and eight studies published between 1984 and 2020, which explored VOCs as cancer biomarkers were subjected to a quantitative analysis. Based on the findings of various studies, the researchers identified and categorized VOCs linked to various types of cancer based on both sampling approaches and analysis methods [18]. They deliberated on potential challenges and opportunities for advancement in cancer research, drawing from their combined analysis [19, 20].

Using both urine and breath samples, the dog identified cancer in 40 out of 41 samples, achieving a 97.6% success rate [19]. The dog exhibited an 87.8% success rate in identifying cancer using urine samples alone. Thirty two out of the 41 cancer breath samples were correctly identified by the dog with a success rate of 78%. Across various studies, detection rates varied significantly.

### Limitations

The study encountered several limitations in its scientometric analysis. In oncology, the number of research studies focusing on canine olfactory detection is limited. More research is needed for a comprehensive analysis and verification of the results. The role of dogs’ olfactory ability in cancer detection warrants further investigation. Scopus boasts a more comprehensive collection of journals and publications, covering most publications relevant to our topic.

## Conclusion

In this study, Guest, Harris, and Lippi significantly advanced canine olfactory detection in oncology. The Journal of Breath Research, PLoS One, and Scientific Reports were identified as significant outlets for publishing research in this field. Applying Lotk’s and Bradford’s bibliometric laws, distinct authorship patterns and journal distribution dynamics have been uncovered. The study period revealed the enduring significance of journals in the Q1 and Q2 quartiles, as evidenced by the CiteScore impact analysis. The global nature of research in this domain is highlighted by the identifications of key contributors from Poland, the UK, and the US. The analysis has identified patterns and key contributors, offering a solid base for future research. The multidisciplinary field necessitates ongoing collaboration and exploration among researchers and institutions.

## Authors’ Contributions

FEC, FMT, and JPM: Searched the database and analyzed the data. DGV, CMV, JM, FM, FEC, and FMT: Wrote the main manuscript text and prepared the table. JPM and FMT: Analyzed the data. FMT, FM, CMV, JM, FEC, DGV, and JPM: Designed the study and critically reviewed and modified the manuscript. All authors have read, reviewed, and approved the final manuscript.
